# DNA methylation reprogramming in medaka fish, a promising animal model for environmental epigenetics research

**DOI:** 10.1093/eep/dvaa008

**Published:** 2020-07-07

**Authors:** Xuegeng Wang, Ramji K Bhandari

**Affiliations:** Department of Biology, University of North Carolina Greensboro, Greensboro, NC, 27412, USA

**Keywords:** epigenetic reprogramming, medaka, embryo, primordial germ cells, DNA methylation

## Abstract

DNA methylation is a major epigenetic modification that undergoes dramatic changes in two epigenetic reprogramming windows during development: first in preimplantation embryos and second in primordial germ cell (PGC) specification. In both windows, DNA methylation patterns are reprogrammed genome-wide, and the majority of inherited methylation marks are erased, generating cells with broad developmental potential. Recent studies reported that the reprogramming of genome methylation in medaka is similar to human and mouse, suggesting that medaka may serve as a suitable biomedical model for comparative studies focused on the epigenetic and transgenerational inheritance of phenotypic traits. In this mini review, we will discuss how somatic and germ cells in post-fertilization stage embryos are epigenetically reprogrammed in mammals and fishes with a particular focus on DNA methylation dynamics.

## DNA Methylation Reprogramming in Somatic Cells during Embryogenesis

The gametes are highly specialized cells. In mammals, after fertilization, both paternal and maternal epigenetic modifications are reprogrammed to totipotent status by the blastula stage. Aberrant epigenetic alterations that occur at this early stage result in differential gene expression across many tissue types and have been described as the potential mechanism behind the developmental origins of health and disease (DOHaD) [1]. The paternal genome undergoes rapid demethylation during the first cell cycle and continues demethylation during cleavage, whereas the maternal genome undergoes the process of gradual demethylation, and the parental nucleus gains the similar methylation pattern by blastula stage [2–4]. In contrast, unlike in the mammals, Jiang *et al.* [5] reported a unique DNA methylation dynamic process in zebrafish (*Danio rerio*). The paternal genome methylation pattern remains stable, and the global methylation of the maternal genome gradually increases. When an embryo develops into the early blastula stage, methylation levels of maternal genome reach the level equivalent to methylation pattern of the paternal genome, suggesting the absence of a mammalian pattern of DNA methylation reprogramming in zebrafish embryogenesis [5]. However, a study by Mhanni *et al.* [6] reveals an interesting DNA methylation pattern in zebrafish fertilized eggs and cells undergoing cleavage that resembles DNA demethylation patterns in mammals and medaka. In their study, relative methylation in the fertilized eggs was observed at the lowest level and gradually increased in the following stages. On the other hand, Jiang *et al.* showed the DNA methylation dynamics surge at 1.16 h (16-cell stage) after fertilization and seemed to have missed this important window of early embryonic development in zebrafish, especially cleavage stages from the 1-cell through the 16-cell stage. The study by Jiang *et al.* was based on a single-base resolution DNA methylation profile, whereas the DNA methylation dynamics observed by Mhanni *et al.* was based on results obtained from restriction digestion with methylation-sensitive enzymes followed by blotting of DNA [5, 6]. Hence, it is essential to reveal a high-resolution profile of epigenetic reprogramming in the post-fertilization stage zebrafish embryo, including the DNA methylation landscape from the 1 cell to the 16 cell stages to clarify this discrepancy.

**Figure 1:**
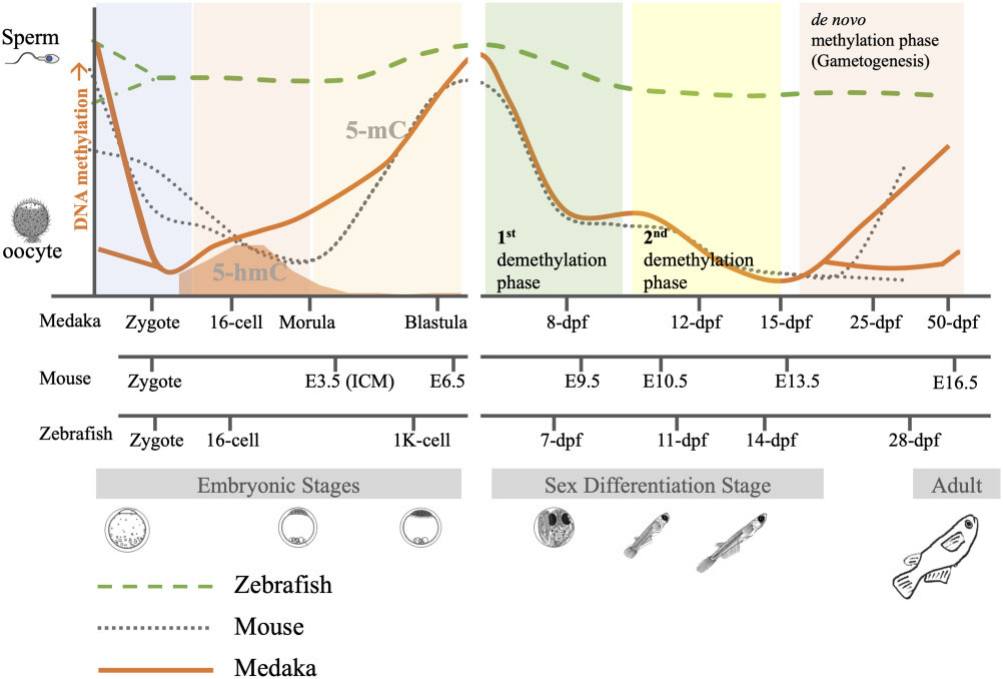
DNA methylation reprogramming model during embryogenesis (left panel) and PGC specification (right panel) in medaka. Left panel: Sperm is hypermethylated, and oocyte is hypomethylated. Paternal genomic methylation is erased in the zygote. Embryos maintain genome hypomethylation during the first several cell cycles. Global DNA methylation levels increase from the 16-cell stage to the Blastula stage. Right panel: In PGCs, the demethylation occurs in two phases: the first phase occurs before 8-dpf (1st demethylation phase), and the second phase occurs between the 10-dpf to the 12-dpf stage (2nd demethylation phase). Global DNA methylation level increases from 15-dpf to 25-dpf in male PGCs, whereas female PGCs remain hypomethylated. Data set sources: medaka embryogenesis [7]; medaka PGCs [13]; mouse embryogenesis [4]; mouse PGCs [9]; zebrafish embryogenesis [5] and zebrafish PGCs [12].

Medaka (*Oryzias latipes*) serves as an excellent animal with several advantages, including genetic sex-determination in the *Hd-rR* strain. A recent study reported that medaka has a DNA methylation reprogramming process comparable to humans and mice (Fig. 1, left panel) [2–4, 7]. In the medaka, the sperm genome is hypermethylated, whereas the oocyte genome is hypomethylated. Medaka embryos erase the paternal genome methylation pattern within the first cell cycle, and then the global DNA methylation levels gradually increase *de novo* from the 16-cell stage to gastrula stages. This observation shows that thesomatic cell reprogramming in medaka fish is comparable to mammals [7].

## DNA Methylation Reprogramming in Primordial Germ Cells

PGCs are the only embryonic cells with the potential to transmit genetic and epigenetic information to the next generation. PGC development is one of the windows of susceptibility to environmental stressors, and epigenetic alterations induced in this stage may result in transgenerational phenotypic traits [8]. PGC demethylation completes in two phases in mice [9]. During Phase 1, the PGCs undergo global demethylation and erase the methylation modifications established in the epiblast (E6.5, embryonic day 6.5). During Phase 2, locus-specific DNA demethylation makes the PGCs hypomethylated at embryonic day E13.5. In humans, DNA demethylation completes in a similar fashion during epigenetic reprogramming in PGCs [10, 11]. In zebrafish, the germline does not undergo genome-wide erasure of DNA methylation during development, indicating the absence of genome-wide DNA methylation reprogramming in zebrafish PGCs [12]. Recent studies by Wang and Bhandari [13] demonstrated that the DNA methylation reprogramming process during PGCs specification in medaka is comparable to mice and humans (Fig. 1, right panel). The reprogramming in medaka completes in two phases. The first global demethylation phase completes before 8-days post-fertilization (dpf), whereas the second demethylation phase starts at 10-dpf and completes by 12-dpf. By 25-dpf, *de novo* methylation initiates in male PGCs but not in female PGCs [13]. It is not clear at the moment whether distinct epigenetic reprogramming in sex cells are linked to presence or absence of genetic sex-determination system as mammals and medaka (Hd-rR strain) have XX/XY genetic sex-determination system [14, 15], while zebrafish lack sex chromosomes and genetic sex-determination [16]. The overall pattern of PGC reprogramming in medaka, which is comparable to mammals, makes medaka an excellent model organism to study the mechanisms underlying intergenerational and transgenerational epigenetic inheritance of phenotypes.

## Medaka Fish as an Animal Model for Studying the Environmentally Induced Epigenetic Inheritance of Traits

Perturbations of DNA methylation reprogramming events have the potential for health problems later in life and adverse health outcomes in descendants. Post-fertilization epigenome reprogramming modulates the outcome of epimutation by erasing epigenetic marks transmitted through gametes. A variety of environmentally induced transgenerational health effects have been observed in both mammals and fish [17–19]. It is not clearly understood whether environmentally induced DNA methylation marks escape the reprogramming events described herein or there are other windows of reprogramming occurring during gametogenesis. Since PGCs have the potential to transmit epigenetic alterations to the offspring, it is critical to illustrate the relationship between epigenetic reprogramming events in PGCS and transgenerational inheritance of environmentally induced epimutations and associated phenotypes in subsequent generations. Given that epigenetic reprogramming during embryogenesis and PGCs specification are similar between mammals and medaka fish, we propose that the following questions be addressed in the future studies using the medaka fish to understand the mechanisms for transgenerational epigenetic inheritance.

1. How do environmental stressors induce DNA methylation marks in PGCs and gametes? The dialogues between environmental stressors and epigenetic modifications on DNA should be addressed at the molecular and biochemical level. Studies have shown that TET proteins play a crucial role in PGC development and are sensitive to environmental stressors [20–22]. Medaka fish have DNA methylation and demethylation related genes homologous to mammals and can be used as a model to investigate the underpinning mechanisms.
2. How do environmental stressor-induced DNA methylation marks escape the global epigenetic reprogramming event? DNA methylation marks must survive in at least two rounds of the reprogramming process in a life history stages of an organism. The high similarity of DNA methylation reprogramming mechanism between medaka and mammals suggests that the medaka fish can serve as an excellent animal model to study epigenetic transgenerational inheritance. Furthermore, mechanisms as to how small RNAs (e.g. miRNAs or tRNAs) establish stressor-specific epigenetic memory in PGCs require further investigation. Medaka express miRNAs in germ cells, so the role of miRNAs can be studied in medaka.
3. What is the relationship between differential DNA methylation marks and the development or progression of adverse phenotypes?. DNA methylation marks are inherited by different cell types and result in altered gene expression across many tissue types. Because of the conserved epigenetic reprogramming between medaka and mammals, the medaka may offer several advantages as an animal model when it comes to addressing mechanisms underlying biological processes concerning PGC development, totipotency, and transgenerational epigenetic inheritance of environmentally induced traits and epigenetic memory. In general, aquarium fish have been demonstrated to be promising animal models for studying the transgenerational inheritance of phenotypes [23, 24], and have advantages over mammalian models due to external fertilization, embryo development, short generation and ease of handling.
